# Biopsy of Different Oral Soft Tissues Lesions by KTP and Diode Laser: Histological Evaluation

**DOI:** 10.1155/2014/761704

**Published:** 2014-10-27

**Authors:** Umberto Romeo, Claudia Russo, Gaspare Palaia, Rossella Lo Giudice, Alessandro Del Vecchio, Paolo Visca, Guido Migliau, Alberto De Biase

**Affiliations:** ^1^Department of Oral and Maxillofacial Sciences, “Sapienza” University of Rome, Via Caserta 6, 00161 Rome, Italy; ^2^Department of Cytology and Cellular Diagnostics, Regina Elena Institute, Via Elio Chianesi 53, 00144 Rome, Italy

## Abstract

*Introduction*. Oral biopsy aims to obtain clear and safe diagnosis; it can be performed by scalpel or laser. The controversy in this latter application is the thermal alteration due to tissue heating. The aim of this study is the histological evaluation of margins of “*in vivo*” biopsies collected by diode and KTP lasers. *Material and Methods*. 17 oral benign lesions biopsies were made by diode 808 nm (SOL, DenMatItalia, Italy) and KTP 532 nm (SmartLite, DEKA, Italy). Samples were observed at OM LEICA DM 2000; margin alterations were evaluated through Leica Application Suite 3.4. *Results*. Epithelial and connective damages were assessed for each pathology with an average of 0.245 mm and a standard deviation of ±0.162 mm in mucoceles, 0.382 mm ± 0.149 mm in fibromas, 0.336 mm ± 0.106 mm in hyperkeratosis, 0.473 mm ± 0.105 mm in squamous hyperplasia, 0.182 mm in giant cell granuloma, and 0.149 mm in melanotic macula. *Discussion*. The histologic aspect of lesions influenced the response to laser, whereas the greater inflammation and cellularity were linked with the higher thermal signs. Many artifacts were also associated to histologic procedures. *Conclusion*. Both tested lasers permitted sure histologic diagnosis. However, it is suggested to enlarge biopsies of about 0.5 mm, to avoid thermal alterations, especially in inflammatory lesions like oral lichen planus.

## 1. Introduction

A biopsy is a diagnostic procedure which consists in taking a tissue fragment to subject it to a histological examination and, therefore, to obtain a diagnosis of certainty that can or cannot confirm the suspicion clinical diagnostic [1].

Biopsies can be classified according to the used material, the clinician timing, the lesion site, and the used technique that can be distinguished in incisional and excisional biopsies. The incisional biopsy involves the removal of a representative portion of the lesion and a portion of healthy tissue adjacent to it [2–4]; while the excisional biopsy consists in the removal of the whole lesion allowing, at the same time, carrying out both a diagnostic and therapeutic procedure [4, 5].

The biopsy is generally indicated for the following:

- recognizing neoplastic, preneoplastic, and other soft tissue diseases;
- identifying the origin of ulcers that do not heal within two weeks;
- defining the nature of lesions that do not regress after therapy;
- removing lesions of the right dimensions and verifying their nature.

Nowadays it is possible to perform oral biopsies using two different tools, the scalpel and the laser.

The scalpel allows obtaining a tissue fragment characterized by the presence of well-defined peri-incisional margins with no structural alterations. However, this surgery always requires anesthesia and sutures, and the operative field is not bloodless.

The laser devices most commonly used in oral soft tissues surgery are the diode (600–980 nm), the potassium titanyl phosphate (KTP, 532 nm), the carbon dioxide laser (CO_2_, 10600 nm), the neodymium-doped yttrium aluminum garnet (Nd:YAG, 1064 nm), and the erbium-doped yttrium aluminum garnet (Er:YAG 2940 nm).

Lasers, used for biopsies execution, have several advantages than the scalpel. In fact, they consent to obtain a good hemostasis, bloodless field and a faster healing, above all during the initial phases [6].

However, due to the thermal effects of the laser, incisional margins of tissue samples can be altered, creating doubts about the effectiveness of this method in the diagnosis of systemic disease [7]. If the use of this tool has many advantages over the cold blade, the risk of jeopardizing the outcome of histological analysis, due to laser thermal effects on peri-incisional area, still raises doubts. Actual scientific literature does not reveal* in vivo* studies concerning the evaluation of peri-incisional biopsy taken with the laser. Pathological tissues “*in vivo,*” compared to those “*ex vivo,*” are characterized by a higher concentration of liquid, lower cell cohesion, and normal or pathological amounts of blood (e.g., in inflammatory or autoimmune diseases).

This consideration could lead to an improvement in cutting ability of the laser that could permit the parameters applied to be reduced with less damage to cut margins but on the other hand to higher local heat buildup with larger thermal artefacts.

The aim of this “*in vivo”* study is to analyze the tissue fragments removed by laser surgery, to assess the epithelial and connective tissue damage caused by its thermal effects.

## 2. Materials and Methods

Seventeen patients (8F/9M), affected by oral benign pathologies, have been subjected to oral excisional biopsy. In some cases, lesions have been treated using an incisional biopsy because of their site or their size. All tissue samples have been removed by the same operator in order to execute a proper biopsy thanks to his experience and knowledge in laser tools and biological tissue characteristics.

Biopsies have been performed using two different wavelengths with the following parameters: diode laser 808 nm (SOL, DenMatItalia, Italy), power: 2 W in CW, fluence: 2400 J/cm^2^, fiber spot: 320 *µ*m; KTP laser 532 nm (SmartLite, DEKA, Italy), power: 1.5 W in PW, fluence: 212 J/cm^2^, fiber spot: 300 *µ*m. Parameters have been selected considering the right execution of the surgical intervention and the patient compliance never exceeding 5 minutes.

Local anesthesia with 1.8 mL of mepivacaine solution (Mepivacaina Pierrel, 30 mg/mL, injection solution 1.8 mL, Pierrel Spa, Milan, Italy) without vasoconstrictor was performed around the area of the lesion before the beginning of each surgical intervention, injecting the solution at a distance of 0,5 cm from lesions margins. The excised lesions size was between 0,5 and 1 cm of diameter. Two mucoceles were taken out by diode laser and one by KTP laser; 5 fibromas were excised by diode; 3 hyperkeratosis lesions were removed by diode and 1 by KTP; the 3 oral lichen planus, the melanotic macula, and the oral giant cell granuloma were removed by diode laser. After surgery, the samples were sent to the pathologist, for the histological evaluation and diagnosis, in a single-blind mode. No suture or medication was applied and the wound was left to heal by secondary intention. All biopsy samples were fixed in a 10% neutral-buffered formalin solution, embedded in paraffin, sectioned, and stained with hematoxylin-eosin for conventional histopathological evaluation.

Tissue fragments were again observed through the use of an optical microscope LEICA DM 2000, 5x and 10x magnification, and thanks to an appropriate software (Leica Application Suite version 3.4) quantitatively and qualitatively marginal alterations, due to the thermal action of lasers, have been evaluated. Quantitative evaluation carried out a measurement in millimeters and statistical analysis was carried out by calculating the arithmetic mean and standard deviation (a measure of the dispersion of data around the expected value) of the different values, while the involvement of epithelial and connective tissue in thermal alterations has been evaluated in the qualitative aspect. In every oral pathology, connective and epithelial damage have been evaluated in terms of charring and coarctation, since in many cases it was impossible to evaluate them separately.

## 3. Results

Follow-up at 7 and 21 days showed a complete recovery of the wound, without any complications or pain.

The presence of peripheral alterations has not influenced histological analysis: for all samples, it was possible to obtain a certainty diagnosis. Histological examination showed three mucoceles, five fibromas, four hyperkeratotic lesions, three oral lichen planus, one giant cells granuloma, and one melanotic macula.

Peripheral damage has been individually evaluated for each disease, considering that the morphological and structural characteristics of the various lesions could strongly influence the tissues response to the laser action.

Graphs have been realized to show the trend of the measures, and statistical analysis has been carried out calculating the mean and standard deviation of the different values. Moreover, in each histological group the same parameters have been evaluated.

**Figure 1:**
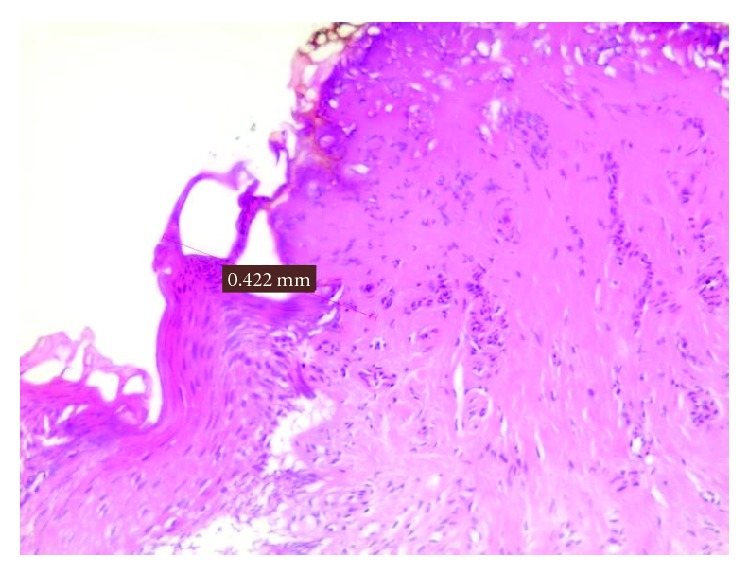
Damage measurement in a mucocele.

**Figure 2:**
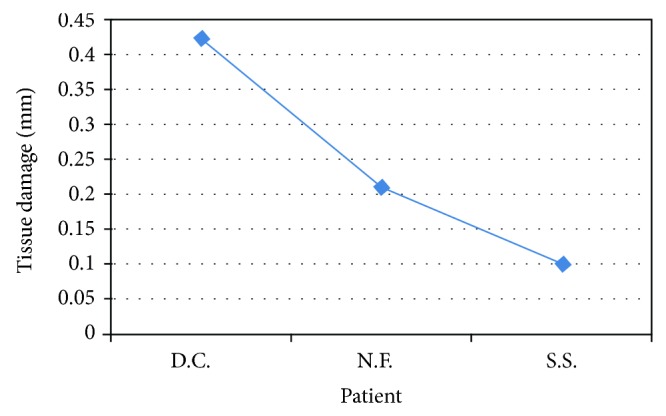
Peri-incisional marginal damage in mucocele.

The histological evaluation of peri-incisional margins in which the microscopic analysis was compatible with the diagnosis of mucocele showed a damage average of 0.245 mm with a standard deviation of ±0.162 mm (Figures 1 and 2; Table 1). Only in one case the epithelium was not visible because the damage was exclusively assessed to the connective tissue.

**Figure 3:**
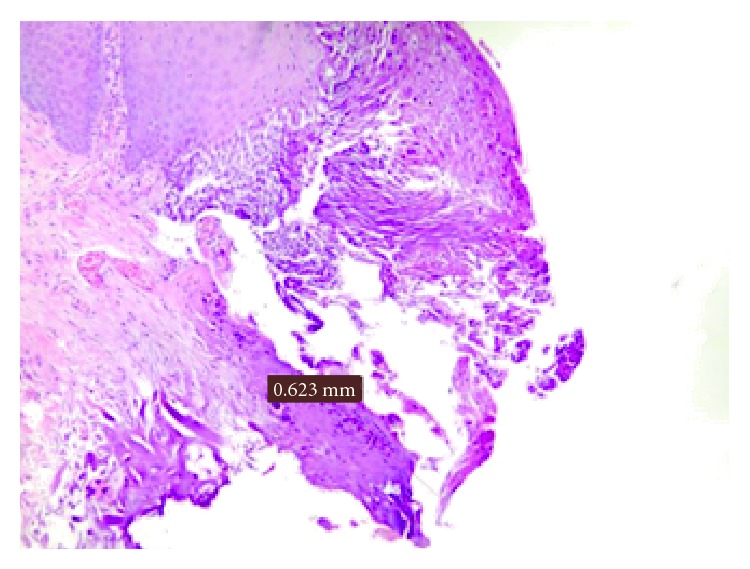
Damage measurement in a fibroma.

**Figure 4:**
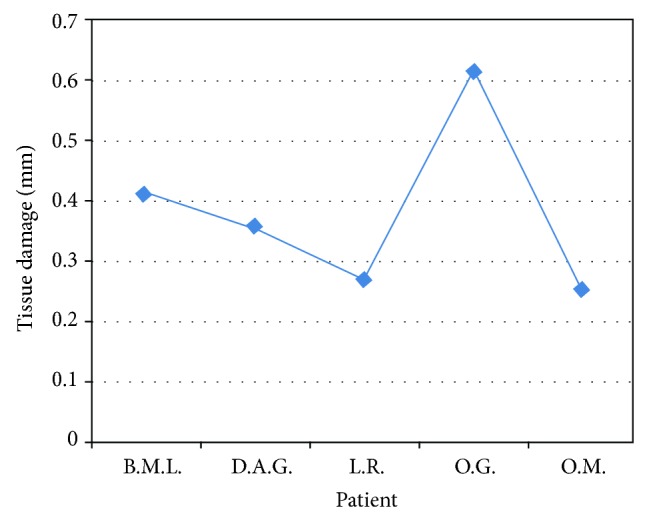
Representation of peri-incisional marginal damage in fibroma.

In the clinical cases in which the histological evaluation leads to diagnosis of fibromas (Figures 3 and 4; Table 2) the damage average was of 0.382 mm and the standard deviation was of 0.149 mm. Also in one of these cases, it was not possible to consider the epithelium of a sample because the damage has been measured only in the connective layer.

**Figure 5:**
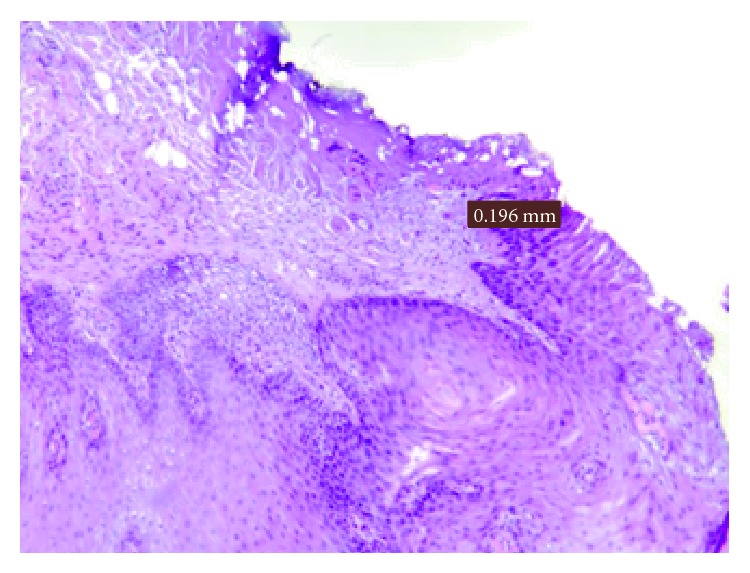
Damage measurement in a hyperkeratotic lesion.

**Figure 6:**
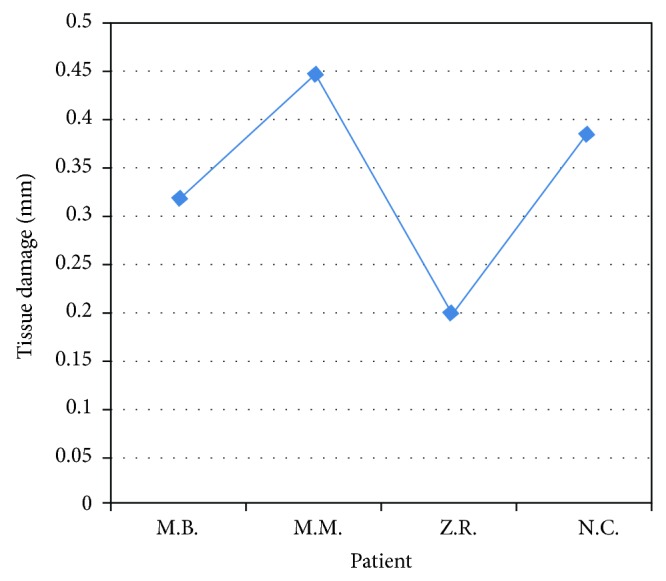
Peri-incisional marginal damage in hyperkeratotic lesion.

Histological evaluation of the peri-incisional margins of hyperkeratotic lesions (Figures 5 and 6; Table 3), compatible with the diagnosis of squamous hyperplasia, showed a damage average of 0.336 mm with a standard deviation of ±0.106 mm.

**Figure 7:**
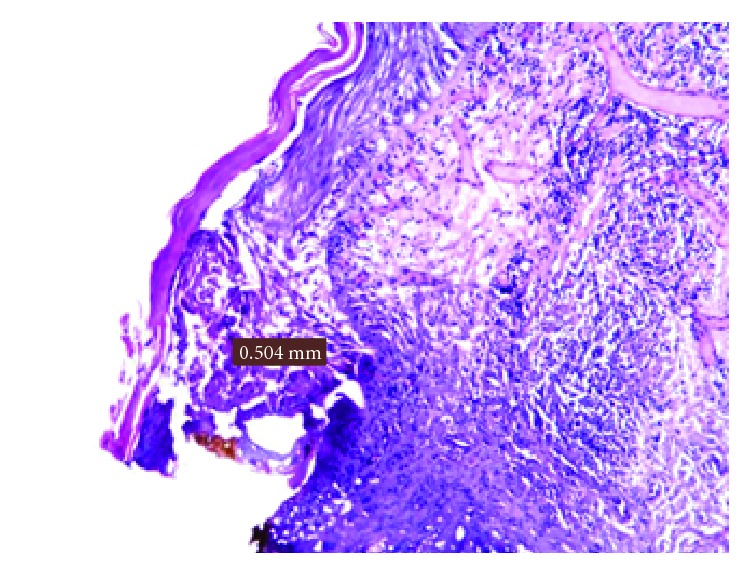
Damage measurement in an oral lichen planus.

**Figure 8:**
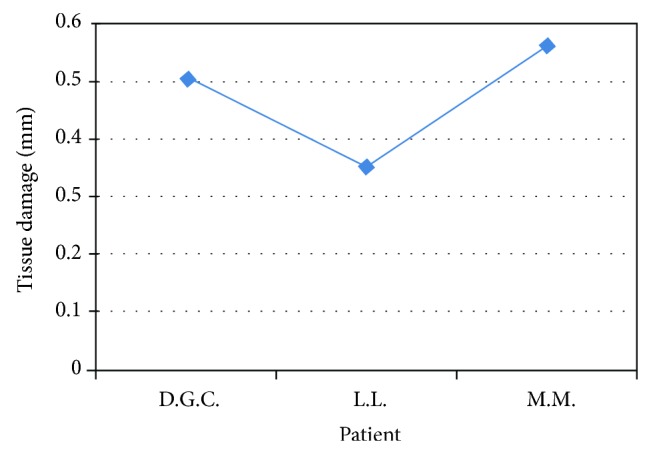
Representation of peri-incisional marginal damage in oral lichen planus.

Furthermore, the histological evaluation of the peri-incisional margins of clinical cases whose diagnosis was oral lichen planus demonstrated a damage average of 0.473 mm with a standard deviation of ±0.105 mm (Figures 7 and 8; Table 4).

**Figure 9:**
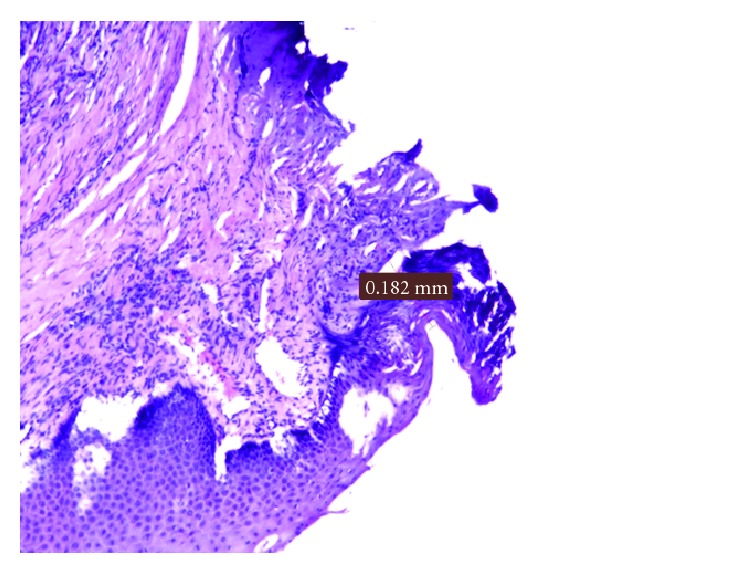
Damage measurement in a giant cells granuloma.

The damage average in the microscopic examination of the giant cell granuloma was 0.182 mm (Figure 9).

**Figure 10:**
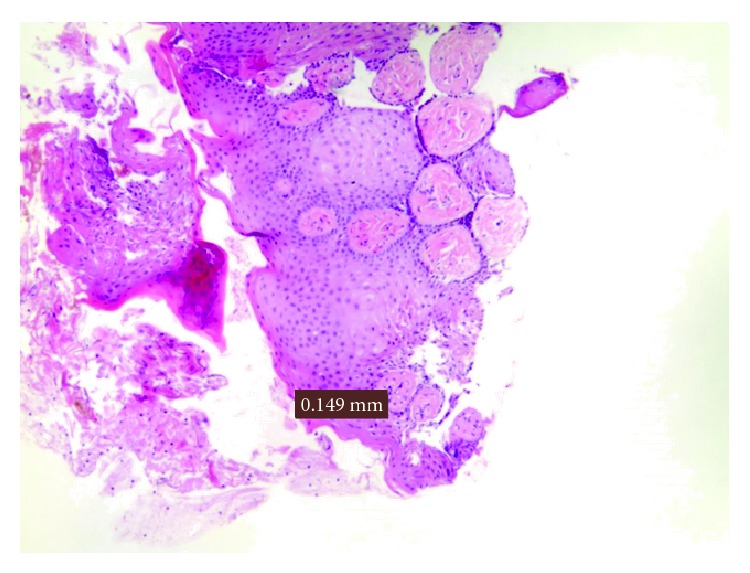
Damage measurement in a melanotic macula.

Finally, in the melanotic macula, removed by the diode laser 808 nm, the damage average was equal to 0.149 mm (Figure 10).

## 4. Discussion

Several studies are present in the literature about [8–11] the use of laser in oral soft tissue biopsy, but only few of them focus on the damage caused by this device at peri-incisional margins of tissue fragments. Every type of laser can create thermal damage to the target tissues because of the photothermal effect. While lasers work, they heat tissues, causing a temperature increase, at the point of incidence, of more than 100 degrees. Surrounding tissues can be involved in the increase of temperature and so they are permanently or reversibly damaged. Furthermore, the histologic exam is linked to the integrity of peri-incisional margins, and this is a basic requirement for a tool employed in biopsies.

A study carried out on rabbits by Rizoiu et al. [11] showed no differences in the histology among peri-incisional margins of samples excised by laser and by scalpel. In a Dalrymple and Russell's study [12], about the evaluation of peri-incisional margins of incisional and excisional biopsies performed by CO_2_ laser on cervix lesions, it appeared that marginal alterations were on average 0.3 mm. However, due to thermal damage in 12% of cases the histological examination gave an uncertain outcome.

In a study carried out by Romeo et al. [13], the effects of Er:YAG, Nd:YAG, Er-Cr:YSGG, and two diode lasers (resp., 808 nm and 980 nm) have been evaluated on pig tongue. It resulted in the fact that each kind of laser device could be used to perform biopsy. Even if they caused slight alterations in the taken tissue margins, no one of them compromised the histological evaluation. In particular, the best results have been obtained with the 808 nm diode laser device in pulsed wave mode and with Er-Cr:YSGG laser at higher power, which created peripheral damage less than 1 mm.

Another study carried out by Romeo et al. [14] about the histological evaluation of Er:YAG laser effect on oral soft tissues showed that, using this device with intermediate power (80–100 mJ), the thermal damage was always under the millimeter involving only the epithelium layer. So, authors concluded that thermal damages was negligible and the readability of the perioincisional margins was always possible.

Moreover, a study about the effects of the KTP [15] on oral soft tissue demonstrated that it allowed the execution of precise cut provoking a minimum cellular damage in the epithelium and in the chorion. The precision of the obtained margins make them similar to those ones obtained through the use of a scalpel. In addition to this, specimens of all tested groups were free from thermal artefacts above all when lowest fluence settings have been used.

Furthermore, in a study carried out by Merigo et al. [16] concerning the use of different wavelengths in laser-assisted surgery, it was shown that positive results have been obtained for the evaluation of laser-excised samples in terms of their readability and diagnostic reliability.

Vescovi et al. [17] performed a preliminary histological analysis of specimens from the human oral mucosa comparing Nd:YAG laser versus traditional scalpel. Epithelial changes, connective tissue modifications, presence of vascular modifications, incision morphology, and the overall width of tissue modification were evaluated. Differences between specimens removed with two different parameters of Nd:YAG (3.5 W, 60 Hz and 5 W, 30 Hz) laser were not significant with regard to stromal changes and vascular stasis. The quality of incision was better and the width of overall tissue injuries was less in the specimens obtained with higher frequency and lower power (group 1: Nd:YAG laser at 3.5 W and 60 Hz).

In a retrospective study, Angiero et al. [18], 608 cases of soft tissue lesions localized in the oral cavity (cheek, gingiva, buccal mucosa, tongue, and lips) were examined. Specimens were excised with an 808 nm diode laser, output 1.6–2.7 W, in continuous-wave mode with fibers of 320 *µ*m. The data for specimens larger than 3 mm excised with the diode laser were not significant in terms of stromal changes or vascular stasis, while epithelial and stromal changes were significantly more frequent in specimens with a mean size below 3 mm. Authors suggest that the specimens taken have “*in vivo*” a diameter of at least 5 mm in order to have a reliable reading of the histological sample, but this recommendation is valid even for a scalpel biopsy.

According to several studies the possibility to evaluate “*in vivo*” the marginal alterations of samples excised by laser is not clear. For this reason, there was a necessity to begin an “*in vivo*” study concerning the histological exam of peri-incisional margins after laser biopsy.

This study showed that the biopsy of oral soft tissues, performed by diode or KTP laser, did not create any significant marginal alterations that could compromise the histological diagnosis. Moreover, it shows, as explained in the previous “*ex vivo”* study [13], that the laser device that causes less thermal damage is the KTP. In general, in fact, the laser tissue interaction is due to the operator-dependent factors (modality of use, application time, and choice of the cutting distance from the lesion margins) and the operator-independent factors related to the wavelength and to the optical properties of the tissue.

The bioptic samples of this study showed that carbonization and coarctation were more limited in specific lesions (mucocele) than in others (oral lichen planus), demonstrating in this way how the increased cellularity and inflammation, typical of some lesions, can cause an increase of the peri-incisional damage.

Moreover, it is important to consider that many of the artifacts, found on the samples, were not due to the action of the laser but due to problems which occurred during the process of fixing, cutting, and staining of the tissue fragment.

Finally, the use of laser devices is not advisable to perform biopsies of suspicious lesions. In this case, the analysis of cellular infiltration in the adjacent tissues is fundamental and the thermal effects of the laser may affect the possibility to realize a proper analysis of the lesion margins and to establish the real cancer size [8, 19, 20].

## 5. Conclusions

Laser devices, used by a skilled operator, allow obtaining histological tissue fragments with important advantages both for the operator and for the patient. In fact, thanks to the laser-haemoglobin interaction, the surgical field is bloodless, permitting having greater visibility and also performing surgery in patients affected by coagulation disorders. Furthermore, it is possible to reduce the amount of local anesthesia and to achieve a faster postoperative healing, especially in the early stages.

In this study, it was always possible to obtain a sure histological diagnosis for each sample.

So, laser devices, because of their excellent surgical properties, can be used successfully to perform oral soft tissues biopsies, but a clinical preliminary analysis of the lesion is fundamental, in order to predict whether the peri-incisional thermal damage will be more or less extended. However, the peri-lesional damage did not compromise the morphological and structural characteristics of the specimens.

## Figures and Tables

**Table 1:** Mucocele biopsies.

Patient	Sex	Lesion site	Laser	Damage (in mm)
D.C.	F	Inferior lip	Diode 808	0,442
N.F.	F	Upper left lip	KTP	0,213
S.S.	M	Inferior lip	Diode 808	0,102

**Table 2:** Fibroma biopsies.

Patient	Sex	Lesion site	Laser	Damage (in mm)
B.M.L.	F	Left cheek	Diode 808	0,411
D.G.	M	Left cheek	Diode 808	0,357
L.R.	F	Left tongue margin	Diode 808	0,267
O.G.	F	Left cheek	Diode 808	0,623
O.M.	M	Right cheek	Diode 808	0,252

**Table 3:** Hyperkeratotic lesion biopsies.

Patient	Sex	Lesion site	Laser	Damage (in mm)
M.B.	M	Right tongue margin	Diode 808	0,319
M.M.	M	Lower lip	Diode 808	0,446
Z.R.	F	Lower left lip	KTP	0,196
N.C.	M	Right cheek	Diode 808	0,383

**Table 4:** Oral lichen planus biopsies.

Patient	Sex	Lesion site	Laser	Damage (in mm)
D.G.C.	F	Right cheek	Diode 808	0,504
L.L.	F	Right cheek	Diode 808	0,356
M.M.	M	Right cheek	Diode 808	0,561
